# Methylation profile in penile carcinoma reveals unique signature relative to surround tissue and HPV status

**DOI:** 10.1186/1753-6561-7-S2-P30

**Published:** 2013-04-04

**Authors:** Hellen Kuasne, Ilce MS Cólus, Hector Hernandez-Vargas, Ariane F Busso, Mateus C Barros-Filho, Fábio A Marchi, Cláudia A Rainho, Gustavo C Guimarães, André L Carvalho, Cristovam Scapulatempo Neto, André Lengert, Fernando A Soares, José Vassallo, Zdengo Herceg, Silvia R Rogatto

**Affiliations:** 1 Department of General Biology, Londrina State University, Londrina, PR, Brazil; 2 CIPE - AC Camargo Cancer Hospital, São Paulo, SP, Brazil; 3 Epigenetics Group, International Agency for Research on Cancer (IARC); Lyon, France; 4 Institute of Biosciences, UNESP, Botucatu, SP, Brazil; 5 Barretos Cancer Hospital, Barretos, SP, Brazil; 6 Department of Pathology, UNICAMP, Campinas, SP, Brazil; 7 Department of Urology, Faculty of Medicine, UNESP, Botucatu, SP, Brazil

## Background

Penile carcinoma (PeCa) is an important public health problem in poor and developing countries. Despite the unpredictable behavior and aggressive treatment, there are few data on molecular and epigenetic mechanisms reported in PeCa. The aim of this study was to identify epigenetic profile in tumors and surrounding tissue as well as to identify molecular markers in PeCa, according to the Papillomavirus (HPV) infection.

## Patients and methods

Paired PeCa and surrounding tissue samples were collected from 16 patients. Twenty tumors were also included. Methylated (*MethylMiner - Invitrogen*) and unmethylated (digested with restriction enzyme *McrBC*) enriched sequences were hybridized in a 244K Human DNA Methylation Microarray platform (Agilent Technologies). This assay interrogates 27,627 expanded CpG islands and 5081 shore CpG island regions. Genomic Workbench Standard (v 5.0.14) and BRB softwares were used to analyze the data.

## Results

It was considered only probes with p value<0.001, FDR <0.05 and located inside or in the gene promoter. HPV positivity was detected in 43% of cases (*Linear Array HPV Test Genotyping - Roche*), mainly for 16 and 18 subtypes. Penile carcinoma displayed unique signature relative to surround tissue, showing 171 probes methylated and 349 unmethylated exclusively in tumor samples. Several probes related to genes involved in *NOTCH* and *WNT* pathways were altered in HPV positive cases.

## Conclusions

It was found a differential methylation profile according to HPV status (positive or negative), indicating at least two disrupted pathways, one related to viral infection and the other associated with transcriptional regulation of stem cells.

## Financial support

FAPESP and CNPq.

